# Bayesian Estimation and Inference Using Stochastic Electronics

**DOI:** 10.3389/fnins.2016.00104

**Published:** 2016-03-18

**Authors:** Chetan Singh Thakur, Saeed Afshar, Runchun M. Wang, Tara J. Hamilton, Jonathan Tapson, André van Schaik

**Affiliations:** Biomedical Engineering and Neuroscience, The MARCS Institute, Western Sydney UniversitySydney, NSW, Australia

**Keywords:** Bayesian inference, spiking neural networks, Hidden Markov models, Sequential Monte Carlo sampling, direct acyclic graph, stochastic computation, probabilistic graphical models, neuromorphic engineering

## Abstract

In this paper, we present the implementation of two types of Bayesian inference problems to demonstrate the potential of building probabilistic algorithms in hardware using single set of building blocks with the ability to perform these computations in real time. The first implementation, referred to as the BEAST (Bayesian Estimation and Stochastic Tracker), demonstrates a simple problem where an observer uses an underlying Hidden Markov Model (HMM) to track a target in one dimension. In this implementation, sensors make noisy observations of the target position at discrete time steps. The tracker learns the transition model for target movement, and the observation model for the noisy sensors, and uses these to estimate the target position by solving the Bayesian recursive equation online. We show the tracking performance of the system and demonstrate how it can learn the observation model, the transition model, and the external distractor (noise) probability interfering with the observations. In the second implementation, referred to as the Bayesian INference in DAG (BIND), we show how inference can be performed in a Directed Acyclic Graph (DAG) using stochastic circuits. We show how these building blocks can be easily implemented using simple digital logic gates. An advantage of the stochastic electronic implementation is that it is robust to certain types of noise, which may become an issue in integrated circuit (IC) technology with feature sizes in the order of tens of nanometers due to their low noise margin, the effect of high-energy cosmic rays and the low supply voltage. In our framework, the flipping of random individual bits would not affect the system performance because information is encoded in a bit stream.

## Introduction

Bayesian systems arrive at decisions by interpreting new observations in view of prior knowledge (O'Reilly et al., 2012). A growing body of evidence suggests that neurons in the nervous system calculate Bayesian posterior probabilities of states and events based on observations provided by sensory neurons (MacNeilage et al., 2008; Angelaki et al., 2009; Bobrowski et al., 2009; Fischer and Peña, 2011; Lochmann and Deneve, 2011; Paulin and Hoffman, 2011; Paulin and van Schaik, 2014; Paulin, 2015). It has also been shown that neuronal spikes in a network can act as Monte Carlo samplers and perform Bayesian inference (Huang and Rao, 2014). Several computational models have been proposed for Bayesian state estimation for arbitrary stochastic non-linear dynamical systems (Gordon, 1993; Chen, 2003; Särkkä, 2013). Sequential Monte Carlo (SMC) is one such online estimation algorithm that estimates the posterior density of state space by implementing the Bayesian recursive equation. However, the SMC technique is computationally expensive for real time applications. The computational complexity of SMC arises from the need to calculate a large number of discrete samples of the posterior, which involve integrations over the high-dimensional space of all possible random variables. Some real time implementations of the SMC have been suggested that use clusters of computers to implement the algorithm (Falcou and Chateau, 2006; Chitchian et al., 2013). These are, however, very expensive solutions in terms of area and computational power. One solution to improve this would be to implement an Application Specific Integrated Circuit. However, today's smallest integrated circuit (IC) technologies are susceptible to transient faults, known as soft errors, due to IC reliability issues, alpha particles, cosmic rays and reduction in noise margins by voltage scaling. This poses enormous challenges in the design of deterministic circuits in nanometer IC technology (Yu et al., 2008).

In this work, we describe two types of Probabilistic Graphical Models (PGMs), namely the Hidden Markov Model (HMM) and the Directed Acyclic Graph (DAG) network, and show how these two popular classes of probabilistic algorithms can be built using the same basic building blocks of stochastic hardware.

A PGM uses discrete data structures to efficiently encode and manipulate probability distributions that involve up to several thousands of variables (Pernkopf et al., 2014). Using the HMM network, we propose a novel neuromorphic framework for Bayesian computation, called the Bayesian Estimation and Stochastic Tracker (BEAST) that utilizes SMC in spiking neural networks. We show how the posterior probability distribution of an HMM can be represented by the mean spike count of a population of neurons. In order to explain the BEAST framework, we use an example proposed by Paulin (2014), which describes how the neurons in the optic lobe of a dragonfly could infer the future location of a fruit fly based on the sensory spikes generated as the fruit fly passes in front of the dragonfly's retina. In the second framework, referred to as the Bayesian INference in DAG (BIND), we discuss how neural circuits can perform inference in a DAG network. A DAG is a directed acyclic graph, i.e., a graph with no path that starts and ends at the same vertex. DAGs are used to encode a priori assumptions about individual variables and among variables in causal structures (Koller and Friedman, 2009). There are many DAG networks reported in the literature. For example, Pradhan et al. (1994) have developed a Computer-based Patient Case Simulation (CPCS) system, which has 448 nodes and 908 arcs. These nodes represent various diseases and predisposing factors or symptoms. Each node in the CPCS graph has four possible values on average. Breese et al. have developed another large DAG system for the diagnosis of efficiency problems for large gas turbines (Breese et al., 1992). Our proposed BIND hardware will allow such large networks to run in real time.

There are some previous publications based on probabilistic computation and pulse based arithmetic. Chakrapani et al. (2007) introduced probabilistic CMOS architectures, where the output of its gate primitives are probabilistic in nature due to the lower supply voltage and the resulting small noise margin. Shanbhag et al. (2010) have developed an Algorithmic Noise-Tolerance (ANT) system, which exploits the statistical nature of application level performance metrics, and matches it to the statistical attributes of the device/circuit behavior. Murray (1989) has developed pulse-based mixed signal neural network circuits. Here, neurons generate pulses (spikes) based on their membrane potential using a voltage controlled oscillator; and these spikes are processed in the spike domain at the postsynaptic terminals using pulse-based arithmetic. Pecevski et al. have also shown how the spiking neurons carry out probabilistic inference through sampling in the general graphical models (Pecevski et al., 2011). In another work, Maass has shown how a network of spiking neurons shares similarities with the Boltzmann machine and how it can be applied in solving constraint satisfaction problems and probabilistic inference (Maass, 2015). These prior works concentrate on building deterministic systems from unreliable parts (Chakrapani et al., 2007; Shanbhag et al., 2010) or building neural network using pulse-based logic (Murray, 1989) or developing probabilistic spiking algorithms, which are not hardware friendly (Pecevski et al., 2011). Vigoda (2003) has shown how analog circuits could perform probabilistic message passing algorithms in binary factor graphs. Analog circuits are, however, technology dependent and not easily portable across different fabrication technologies. Although they are suitable for smaller systems, it is difficult to build large systems using analog circuits, as they are not programmable. In addition, the design and testing of large analog systems is difficult compared to digital systems because of the absence of standard design and test flows. Furthermore, there are no standard compilers to convert a model into analog circuits. Our implementations instead build fundamentally probabilistic models for computation using standard logic gates using state-of-the-art ASIC flow.

Here, we use Stochastic Computation (SC) as proposed by Gaines (1969) for the hardware implementation of our proposed Bayesian frameworks. A similar SC-based approach has been described previously by Mansinghka et al. to implement Markov Chain Monte Carlo algorithms in hardware (Mansinghka et al., 2008). In the SC framework, numbers are represented by bit-streams that can be processed using simple digital circuits, and these numbers are interpreted as probabilities. Standard computers are based on deterministic Boolean circuits that simulate propositional deduction according to Boolean algebra, while stochastic algorithms for solving inference under uncertainty are best explained with probability theory. The implementation of probabilistic algorithms on deterministic computers has disadvantages such as the inability to exploit parallelism of the algorithm, and inefficiency in terms of computation time and memory usage. We have employed simple logic gates to implement complex probabilistic algorithms using the SC approach. Our work shows how to build Bayesian computing machines using standard digital logic gates as stochastic computational primitives.

## Materials and methods

In this section we first describe the theory behind our approach (2.1) followed by a section on the implementations (2.2). In the theory section, we first describe the principle of Bayesian inference in an HMM. We then explain the BEAST and the BIND frameworks. In the implementation section, we discuss some canonical neural circuits, which serve as building blocks for the implementation of the BEAST and BIND frameworks in hardware. We then discuss the hardware implementation of inference and learning in the BEAST framework, and inference in the BIND framework.

### Theory

#### Bayesian inference in hidden Markov models

**Figure 1:**
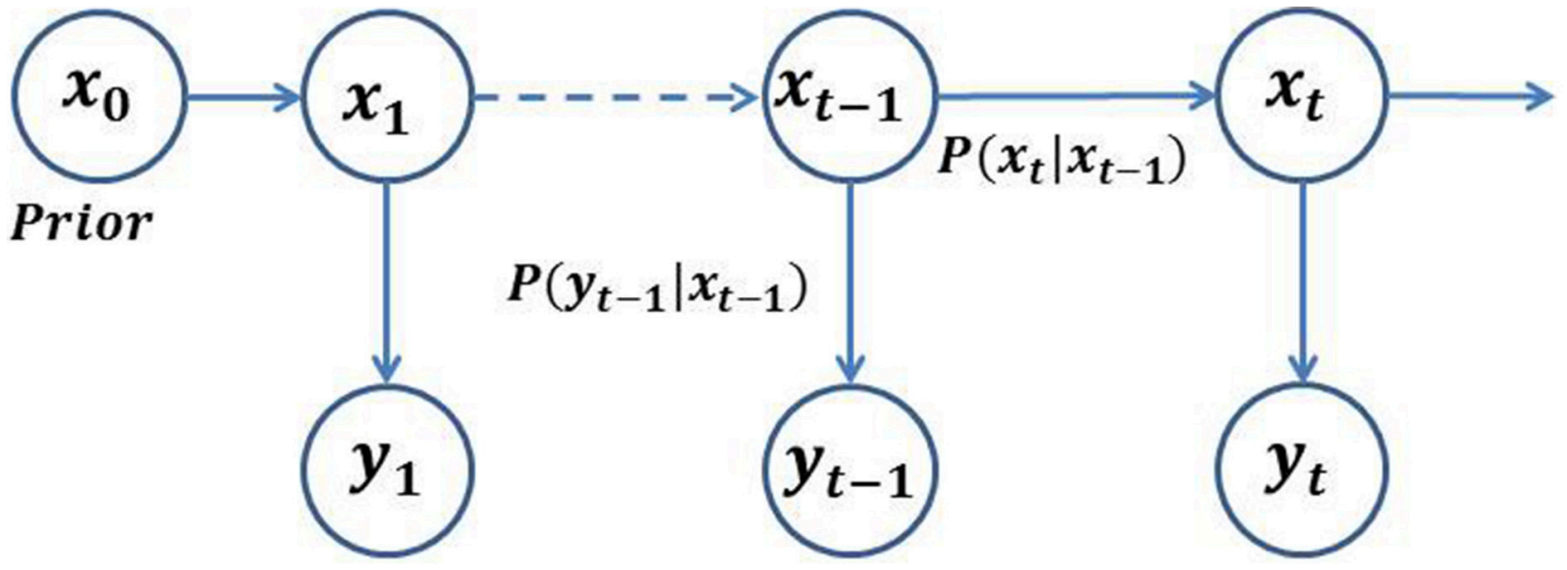
**General architecture of an HMM**. The random variable *X*_*t*_ is the hidden state at time *t*. The random variable *Y*_*t*_ is the observation at time *t*. The arrows in the diagram denote conditional dependencies.

An HMM is a statistical tool for modeling a system characterized by a process that itself is unobservable but generates an observable sequence that depends on the underlying process. In other words, it is a Markov process split into an observable component and an unobservable or “hidden” component (Rabiner, 1989). The unobserved component, *X*_*t*_, may be referred to as the signal process, and the observed component, *Y*_*t*_, as the observation process. In a first order HMM, the evolution of a hidden state depends only on its current state. The observation, *Y*_*t*_, is a noisy function of *X*_*t*_ only. *X*_*t*_ may be estimated by constructing its posterior distribution based only on the noisy measurements or observations, *Y*_*t*_. For a discrete-time estimation problem, the components of the first order HMM may be represented as:

(1) $$\begin{array}{l} X_{t}=f\left(X_{t-1},d_{t}\right) \end{array}$$

(2) $$\begin{array}{l} Y_{t}=g\left(X_{t},v_{t}\right) \end{array}$$

where, *d*_*t*_ and *v*_*t*_ represent random noise sequences with unknown statistics in the discrete-time domain. The state Equation (1) characterizes the state transition model, P(*X*_*t*_|*X*_*t*−1_), whereas equation (2) characterizes the observation model, P(*Y*_*t*_|*X*_*t*_). In a special case, where *f* and *g* are linear and *d*_*t*_ and *v*_*t*_ are Gaussian, a closed loop analytical solution can be obtained, which is known as the Kalman filter (Kalman, 1960). The graphical model in Figure 1 illustrates the stochastic filtering problem described as a generic state-space model. Given an initial prior model, P(*X*_0_); a transition model, P(*X*_*t*_|*X*_*t*−1_); and an observation model, P(*Y*_*t*_|*X*_*t*_); the objective of the filtering is to optimally estimate the current state at a time *t*, given the observations up to time *t*, which amounts to estimating the posterior density, P(*X*_*t*_∕*Y*_1:*t*_). The posterior density function (PDF) may be obtained recursively in two stages—(i) prediction, and (ii) update. In the prediction stage, the next state is predicted from the current state using the state transition model, without using any new observations. In the update stage, the predicted state is corrected using the new observations at time, *t*.

*Prediction stage:*

$$\begin{array}{l} P\left(X_{t}|Y_{1:t-1}\right)=\underset{X_{t-1}}{\sum }P\left(X_{t},X_{t-1}|Y_{1:t-1}\right) \end{array}$$

Using the marginalization principle we obtain:

(3) $$\begin{array}{l} P\left(X_{t}|Y_{1:t-1}\right)=\underset{X_{t-1}}{\sum }P\left({X_{t}\;|\;X_{t-1},\;Y}_{1:t-1}\right)P\left({X_{t-1}|\;Y}_{1:t-1}\right) \end{array}$$

Since *X*_*t*_ does not depend on any past observations, the first term on the RHS of Equation (3) is independent of *Y*_1:*t*−1_ so that we can write:

$$\begin{array}{l} P\left(X_{t}|Y_{1:t-1}\right)=\underset{X_{t-1}}{\sum }P\left(X_{t}\;|\;X_{t-1}\right)P\left({X_{t-1}|\;Y}_{1:t-1}\right) \end{array}$$

where, P(_*X*_*t*_| *Xt*−1_) is the transition model.

*Update stage*:

$$\begin{array}{l} P\left(X_{t}|Y_{1:t}\right)=\;P\left(X_{t}|Y_{t},\;Y_{1:t-1}\right) \end{array}$$

Using the marginalization principle again we get:

(4) $$\begin{array}{l} P\left(X_{t}|Y_{1:t}\right)=\frac{P\left(Y_{t}\;|\;X_{t}\right)P\left({X_{t}|\;Y}_{1:t-1}\right)}{\sum_{X_{t}}P\left(Y_{t}\;|\;X_{t}\right)P\left({X_{t}|\;Y}_{1:t-1}\right)}\; \end{array}$$

where, P(*Y*_*t*_ |*X*_*t*_) is the observation model, and P(_*X*_*t*_| *Y*1:*t*−1_) is as calculated in the prediction stage. For new data observed at time *t*, the new information is used in the update stage using Equation (4).

#### Bayesian estimation and stochastic tracker (BEAST) framework

**Figure 2:**
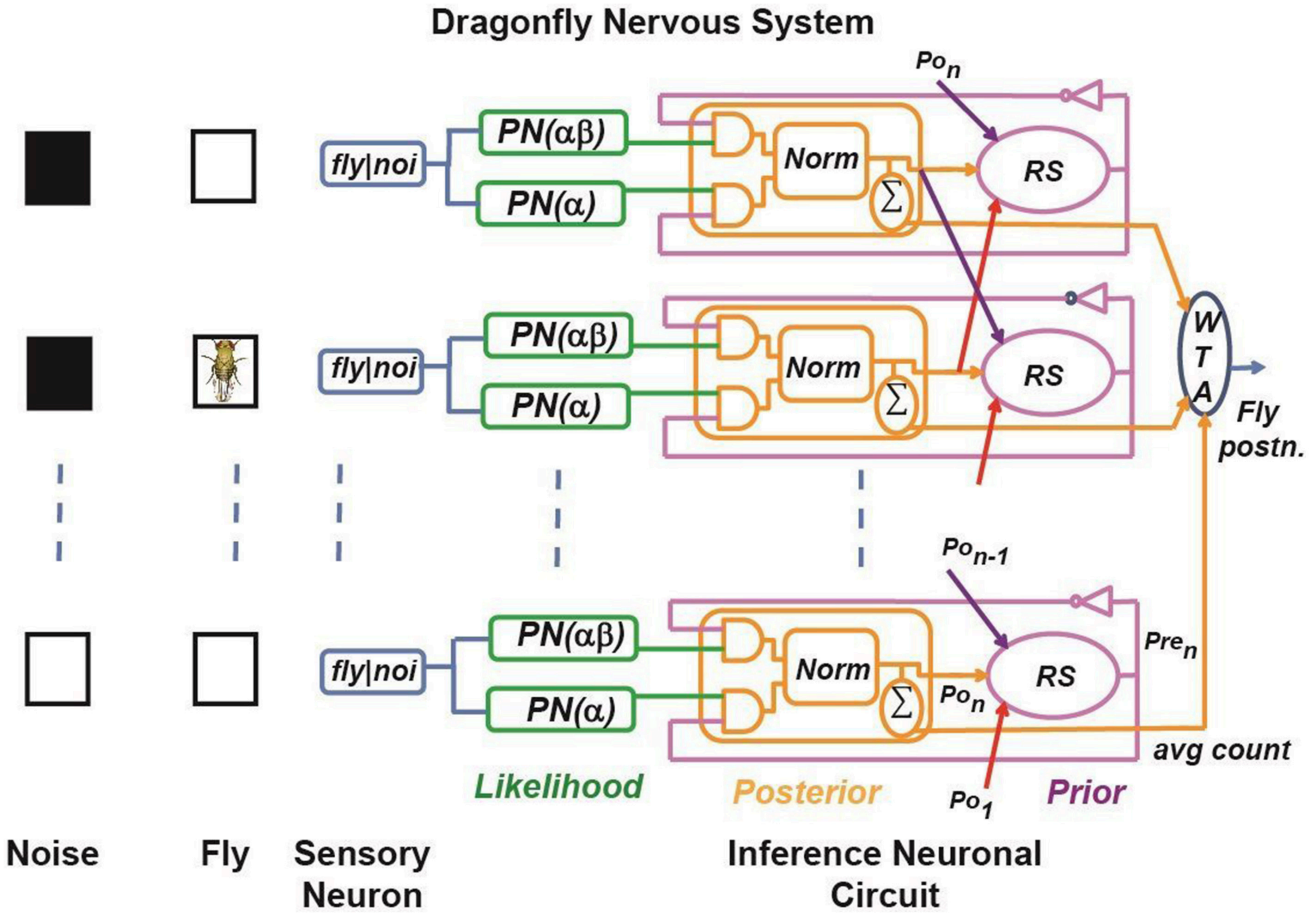
**Simplified model of target tracking in a cluttered environment**. A dragon fly tracks a fruit fly moving against a randomly flickering background. The sensory afferent neurons of the dragonfly fire probabilistically if there is a fruit fly in the foreground, or a false target (*black box*) in the background of their receptive field. Sensory neurons are implemented using a Poisson spike generation block (PN block). The inference neuron population computes the posterior probability distribution by multiplying the sensory neuron output and the prior probability using a coincidence detector (implemented using an AND gate) and by normalizing the product using a normalization circuit, which is shown as *Norm*. The prior probability of each state is calculated by sampling from the adjacent neuronal population posteriors according to the transition probabilities. This is shown as the *RS* (resampling) block. The *WTA* block represents a winner-take-all circuit, which determines the maximum of the posterior distribution across positions to estimate the fly position.

A simplified schematic of our BEAST framework for a dragonfly observing a fruit fly is illustrated in Figure 2. It is a Bayesian model that mimics a simplified neural system of a dragon fly, as proposed by Paulin (2014). Here, a fruit fly moves against a randomly flickering background. The sensory afferent neurons in the optical lobe of the dragonfly fire probabilistically if there is a fruit fly in the foreground, or if there is a false target (distractor) in the background of the dragonfly's receptive field. We have simplified the BEAST framework by introducing two constraints:

- There will be only one fly in the field of vision of the dragonfly at any time.
- The fruit fly has only one degree of movement (one dimensional motion), and it can move only one position to the left, one position to the right, or stay at the same location.

In Figure 2, the first layer represents the sensory afferent neurons, and each sensory neuron is connected to two Poisson neurons (PN). Each PN generates spike trains of a firing rate determined by the presence or absence of spike from the sensory neuron. The firing rates of the PN neurons are functions of the transition probability and the probability of the background noise and these are learned in our model. The next layer represents the inference neuronal circuits, which consist of three different types of sub-circuits named according to their functionality as the coincidence detector (CD) neuron, the normalization (*norm*) neural circuit, and the resampling (RS) neuron. The CD neuron fires a spike when all of its presynaptic neurons fire at the same time. The *norm* neural circuit performs normalization of the firing rate of its presynaptic neurons in the spike domain. The RS neuron is connected spatially to its neighbors, and reroutes the spikes based on the learned transition probabilities. These sub-circuits are described in further detail in Section Implementation. In our model, we assume that the response time of the dragonfly is faster than the time taken by the fruit fly to move a maximum of one step in one-dimensional space. Thus, each RS neuron is spatially connected only to the *norm* neural circuits of the neighboring receptive fields and its own receptive field. For simplicity, let us consider that the receptive field of the dragonfly has *M* sensory neurons, which will divide the state space (*X*_*t*_) into *M* discrete states reflecting the fly's position at time *t*. Each discrete state is associated with a sensory neuron and an inference neuronal circuit. The task of the dragonfly's central nervous system is to predict the fruit fly's position in its receptive field at time *t* by using the statistics of spikes generated by the sensory afferent neurons until time (*t* − 1), and to update the prediction when it gets a new observation, (*Y*_*t*_), at time *t*. The probabilities relevant to the BEAST framework are:

- The probability of background activity, i.e., the presence of a distractor in position *k* (*b*_*k*_ = 1), is P(*b*_*k*_) = β
- The probability of firing of the *k*th sensory neuron, either due to a fruit fly or a distractor is P(*S*_*k*_|*f*_*k*_, *b*_*k*_) = α
- If there is a fly in the receptive field of the *k*th sensory neuron, then it will fire with a probability α, independent of what is in its receptive field background. The likelihoods for the fly in this state, therefore, are:
  $$L_{1k}\;=\;P\left(S_{k}|f_{k}\right)=\{\begin{array}{c} \alpha \;if\left(spike\;if\;fly\;at\;k\right)\\ 1-\alpha \;else\;(no\;spike\;if\;fly\;at\;k\;)\; \end{array}$$
- If there is no fly in the *k*th sensory neuron's receptive field, then there is a distractor in the receptive field with probability β. The marginal likelihoods for no fly in this state are:
  $$L_{0k}=P\left(S_{k}|\tilde{f_{k}}\right)=\{\begin{array}{l} \alpha \beta \;if\;(spike\;when\;no\;fly,\;but\\ distractor\;at\;k)\;\\ 1-\alpha \beta \;else\;(no\;spike\;when\;no\;fly\\ \;but\;distractor\;at\;k)\; \end{array}$$
  Since the dragon fly has no way of knowing whether a sensory spike has resulted from a fly or from background activity, both likelihoods are used each time a spike is received.
- We can estimate the fly's position at time *t* using the HMM framework. Using prediction and update according to Equations (3) and (4), we can write:
  $$\begin{array}{l} P\left(f_{t}|S_{1:t}\right)\propto \;P\left(S_{t}\;|\;f_{t}\right)\underset{X_{t-1}}{\sum }P\left(f_{t}\;|\;f_{t-1}\right)P\left({\;f_{\text{t}-1}|S}_{1:\text{t}-1}\right) \end{array}$$
  where, P(*S*_*t*_ |*f*_*t*_) is the likelihood, P(*f*_*t*_ |*f*_*t*−1_) is the transition probability, and P(_*f*_*t*−1_|*S*1:*t*−1_) is the posterior at the previous time step. Here, *S* ∈ *R*^*M*^, where *M* is the total number of states.

At each time step, we calculate the probability of the fly for each state and a winner-take-all (WTA) circuit determines the maximum a posteriori of the probability distribution across states to estimate the fruit fly position.

#### Bayesian inference in DAG (BIND) framework

**Figure 3:**
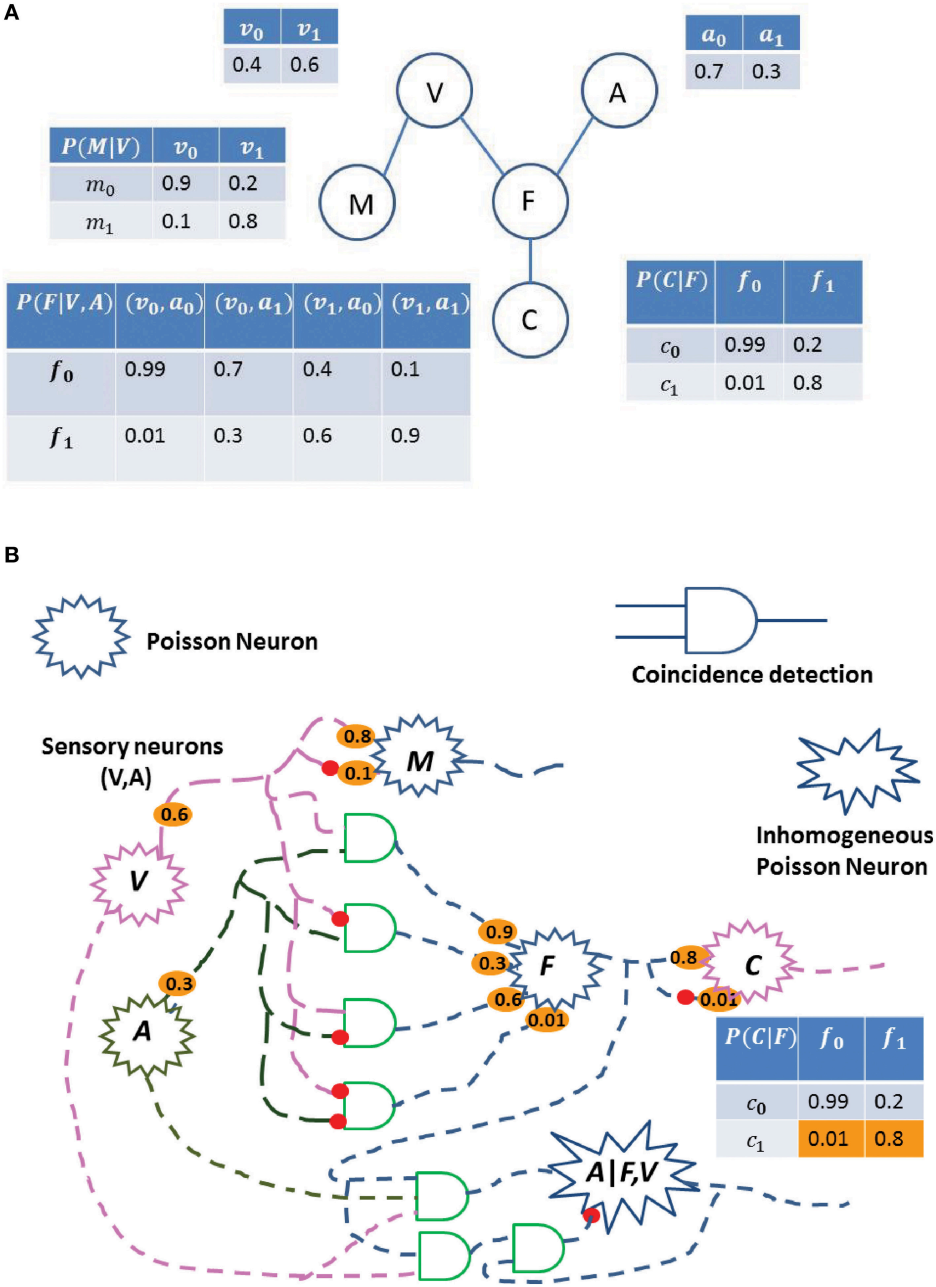
**Probability estimation in a simplified Bayes network. (A)** A predator uses visual cues (V) to find a mate (M), and both visual (V) and auditory (A) cues to find food (F). C is the event of catching the food, F. Each table represents the probability of the event. Some of the events are conditional on other events, such as event (F) depends on (V) and (A). The subscript 0 with the variable represents the probability of the absence of the event, while the subscript 1 represents the probability of the event. For an instance, *P*(*f*_1_|*v*_0_, *a*_1_) = 0.3 means that the probability of finding the food given the auditory cue and absence of the visual cue is 0.3. **(B)** Possible neural circuit corresponding to the Bayes network shown in **(A)**. Each Poisson neuron represents a different random variable (Visual, Audition, Food, Catch, and Mate), and each synaptic connection is associated with the emission probabilities of a spike [non-spiking probability is 1-(spike probability)] shown in orange, and is related to the probability tables of (Figure 6). The probability of a variable can be inferred from the multivariate joint distribution by taking multiple samples and considering only samples when spike occurs for that variable. By the law of large numbers, the estimation of the variable will be close to the true value. For an instance, circuit to compute the probability of variable A given F and V is shown.

As a second example of using stochastic electronics for Bayesian Inference, we now demonstrate how spiking neurons can perform inference in a DAG network. In the BIND framework, we postulate a neural circuit for estimating the probability of an event, based on multiple cues. As an example, consider a simple Bayes network (Figure 3A), where a predator uses visual (V) and auditory cues (A) to find its prey (denoted as Food, F), with a probability distribution of *P*(*F*|*V, A*). The event of catching the food, C, has a conditional probability distribution of *P*(*C*|*F*). Another event, finding a mate (M), depends on the fitness of the predator and is determined only by the visual cue (V), with a conditional probability of *P*(*M*|*V*). Here, we consider each random variable as binary, but the approach can be generalized to use variables of multiple discrete values. In Figure 3A, we have defined the various probabilities associated with the different variables to demonstrate a simple DAG network. The probability of a variable can be inferred from the multivariate joint distribution by counting the number of samples of the variable in a relevant time span. In the described network, we sample each variable in a topological order. We describe the steps below to show how this stochastic sampling method (SSM) works and how a sample can be obtained:

1. Sample *V* from *P*(*V*) (say we get *V* = 1)
2. Sample *A* from *P*(*A*) (say we get *A* = 0)
3. Sample *F* from *P*(*F*|*V* = 1, *A* = 0) (say we get *F* = 1)
4. Sample *M* from *P*(*M*|*V* = 1) (say we get *M* = 1)
5. Sample *M* from *P*(*C*|*F* = 1) (say we get *C* = 0)

Now, we have a sample of the joint distribution (*V* = 1, *A* = 0, *F* = 1, *M* = 1, *C* = 0). Similarly, we repeat the above steps multiple times to obtain many samples. By the law of large numbers, the actual probability of the variable in our samples will converge to the true value. After sufficient samples are obtained, the probability of any random variable, for example, *P*(*A*|*F, V*) can be calculated as:

$$\begin{array}{l} P\left(A|F,V\right)=\frac{\#samples\;with\;A=1,\;when\;\left(F=1\&V=\;1\right)}{\#\;samples,\;when\;\left(F=1\&V=\;1\right)} \end{array}$$

### Implementation

#### Neuronal building blocks

In this section, we discuss the computational elements that we have used to implement the proposed BEAST and BIND frameworks. These neural circuits can be used to build massively parallel, low precision circuits to solve the Bayesian inference problem. Additionally, they provide the possibility of carrying out complex computations with simple hardware, and offer a number of benefits over other computing techniques, such as using only very little silicon area, allowing simple communication over one wire per signal, and a simple implementation. These neural circuits also allow parallel hardware implementations, thus increasing the computational speed. In this work, we have used a bit width of 8 for all the parameters and the variables.

##### Poisson neuron

**Figure 4:**
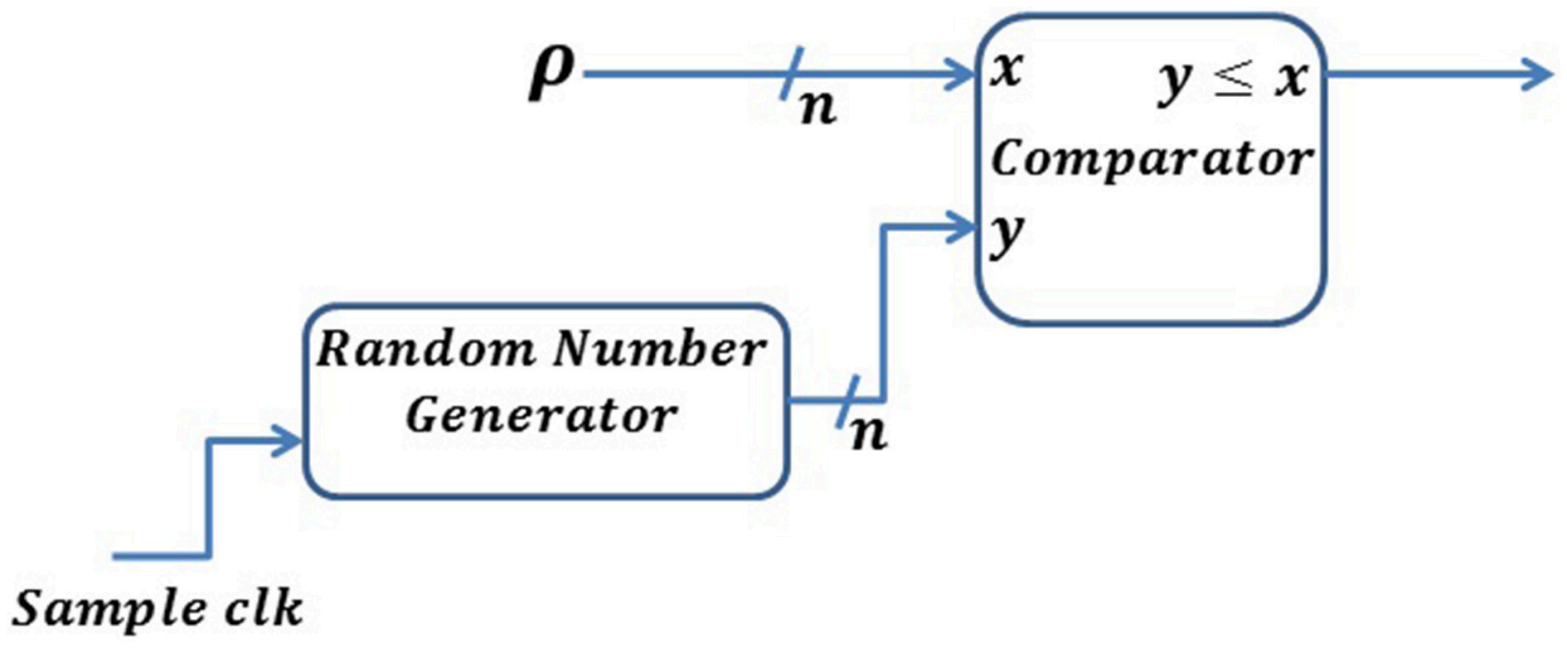
**Digital implementation of a Poisson neuron model**. An *n*-bit random number is generated for each sample *clk* and compared with the spike rate defined by ρ. A spike will be generated whenever the random number is lower than or equal to ρ.

**Figure 5:**
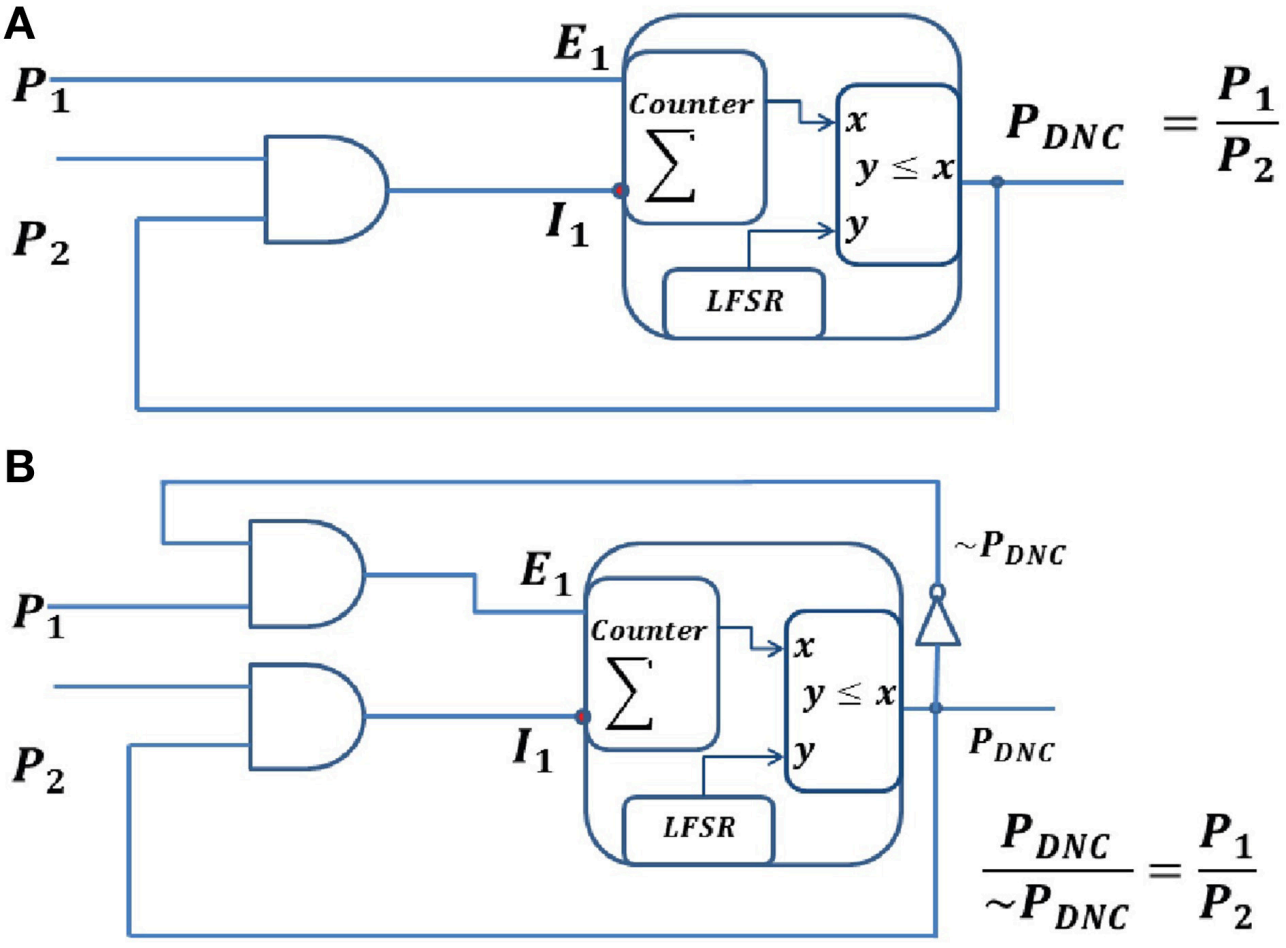
**Division neural circuits for spike train inputs, ***P***_**1**_ and ***P***_**2**_. (A)** Division neural circuit for *P*_1_ ≤ *P*_2_ **(B)** Division neural circuit to represent the division of *P*_1_ and *P*_2_ expressed as the ratio of the two output lines. It can be used irrespective of whether *P*_1_ is less than, equal to or greater than *P*_2_.

In a Poisson neuron (PN) model, the generation of each spike depends only on the firing rate of the neuron and each spike is independent of all the other spikes. In a PN model, the firing rate of a neuron is proportional to the membrane potential above some threshold (Heeger, 2000). We can implement this by generating a sequence of random numbers, *r*_*i*_, uniformly distributed between 0 and 1. For each time, if *r*_*i*_ ≤ ρ, a spike is generated, otherwise no spike is generated. Here, ρ represents the firing rate. Since our implementation uses digital logic gates, we represent probability with a precision of *n* bits. This will map the probability between [0, 2^*n*^−1], as shown in Figure 4. Streams of random numbers are generated by an *LFSR* (Linear Feedback Shift Register) circuit (Golomb et al., 1982). Additionally, we propose an Inhomogeneous Poisson Neuron (IPN), where the output spike rate is time-varying due to a change in the membrane voltage by presynaptic spikes (Figure 5).

##### Coincidence detector (CD) neuron

In the nervous system, a neuron can operate in two distinct ways depending on its synaptic time constant. If the synaptic time constant is longer than the mean inter-spike interval, the neuron acts as a temporal integrator. In contrast, a neuron acts as a coincidence detector if its synaptic time constant is shorter than the inter-spike interval (König et al., 1996). Various functions of the nervous system such as Hebbian learning, binaural localization, and visual attention explain the importance of coincidence detection (Jeffress, 1948; Attneave and Hebb, 1950; Singer and Gray, 1995). For the synchronous hardware implementation of our BEAST model, we have chosen an abstract model for the coincidence detector (CD) neuron and we have implemented it using a simple *AND* logic gate.

##### Division and normalization neural circuit

Normalization (and division) has been suggested to be a canonical neural computation in sensory systems (Carandini and Heeger, 2012), and is thought to bring multiple functional benefits to the neural computations. Evidence suggests that in the olfactory system of invertebrates, GABA (Gamma Aminobutyric Acid)-mediated inhibition is responsible for the normalization operation (Olsen et al., 2010). In the retina, normalization circuits adjust the gain of the neural responses to efficiently use the available dynamic range (Carandini and Heeger, 2012). Normalization circuits in the antennal lobe of the fruit fly are thought to enable odorant recognition and discrimination regardless of concentration (Olsen et al., 2010). Also, normalization has been proposed to account for key empirical principles of multisensory integration, where two sensory inputs interact to modify neural responses (Ohshiro et al., 2011).

A Division Neural Circuit (DNC) computes the division of the firing rate of the input neurons in the spike domain. Figure 5 shows a DNC, which generates spike trains with a rate equal to the division of the two input firing rates. The DNC is a combination of an IPN and a CD neuron. The DNC has one excitatory input, *E*_*i*_, and one inhibitory input, *I*_1_, coming from a CD neuron. This CD neuron receives spike inputs from the output of the DNC (recurrent connection) and from the presynaptic neurons (forward connection). The membrane voltage (ρ) of a DNC is integrated using a saturating counter with 2^*n*^ states, which increases by one unit upon receiving an excitatory spike, and decreases by one unit upon receiving an inhibitory spike. The DNC generates a spike based on its instantaneous membrane voltage (ρ) at each time step. At each time in the output sequence, an *n*-bit random number (*r*) is generated, and if *r* ≤ ρ, then a spike is generated, otherwise no spike is generated. We can write the equations for the DNC as below:

$$\begin{array}{l} E_{1}=P_{1};\;E_{2}=P_{2} \end{array}$$

where, *E*_1_ and *E*_2_ represent excitatory synaptic inputs to the DNC, and *P*_1_ and *P*_2_ represent the firing rates of the presynaptic neurons. The output spike from the DNC and the presynaptic spike are independent of each other, thus the average firing rate of the CD neuron would be the multiplication of the firing rate of its inputs.

$$\begin{array}{l} I_{1}=P_{DNC}\;*\;P_{2} \end{array}$$

where, *I*_1_ is the output of the CD neuron and represent inhibitory connections to the DNC. *P*_*DNC*_ is the output firing rate of the DNC. The membrane voltage is represented as a counter, which acts as an integrator for incoming excitatory and inhibitory spikes. The output of the counter *P*_*DNC*_ can be written as:

$$\begin{array}{l} P_{DNC}=\int P_{1}-P_{DNC}\;*\;P_{2}\\ {\dot{P}}_{DNC}=\;P_{1}-P_{DNC}\;*\;P_{2} \end{array}$$

At equilibrium, the change in output probability is zero, i.e., ${\dot{P}}_{DNC}\;=0,$ which means excitatory and the inhibitory inputs from a neuron are equal. Thus,

(5) $$\begin{array}{l} E_{1}=I_{1}\\ P_{1}=P_{DNC}\;*\;P_{2}\\ {\;P}_{DNC}=\frac{P_{1}}{P_{2}}\; \end{array}$$

The output of the DNC thus computes the division of the firing rate of its inputs as demonstrated in Equation (5), as long as *P*_1_ and *P*_2_ vary more slowly than the time it takes for the DNC to reach its equilibrium state. This circuit is only suitable for *P*_1_ < *P*_2_ and will saturate otherwise, since the stochastic building blocks cannot represent numbers larger than 1. The feedback from the output node to the CD neuron generating *I*_1_ ensures that, even if *P*_2_ is larger than *P*_1_, the membrane potential cannot go below 0. Fortunately, in probabilistic computation, all the variables are in the range of [0,1] and the circuit is thus appropriate for probabilistic computation.

To deal with the case where *P*_1_ > *P*_2_, we need to represent the output signal using two lines ~*P*_*DNC*_ & *P*_*DNC*_. The existing circuit can then be modified by connecting ~*P*_*DNC*_ and *P*_1_ using an AND gate to create the excitatory input (Figure 5B). In this case,

$$\begin{array}{l} E_{1}={\sim P}_{DNC}\;*\;P_{1}\\ {Ṗ}_{DNC}=\;{\sim P}_{DNC}\;*\;P_{1}-P_{DNC}\;*\;P_{2} \end{array}$$

At equilibrium, the change in the output probability is zero, i.e., ${\dot{P}}_{DNC}\;=0,$ which means that the excitatory and the inhibitory inputs of the counter are equal. Thus,

$$\begin{array}{l} {\sim P}_{DNC}\;*\;P_{1}=P_{DNC}\;*\;P_{2}\\ \frac{P_{DNC}}{{\sim P}_{DNC}}=\frac{P_{1}}{P_{2}} \end{array}$$

**Figure 6:**
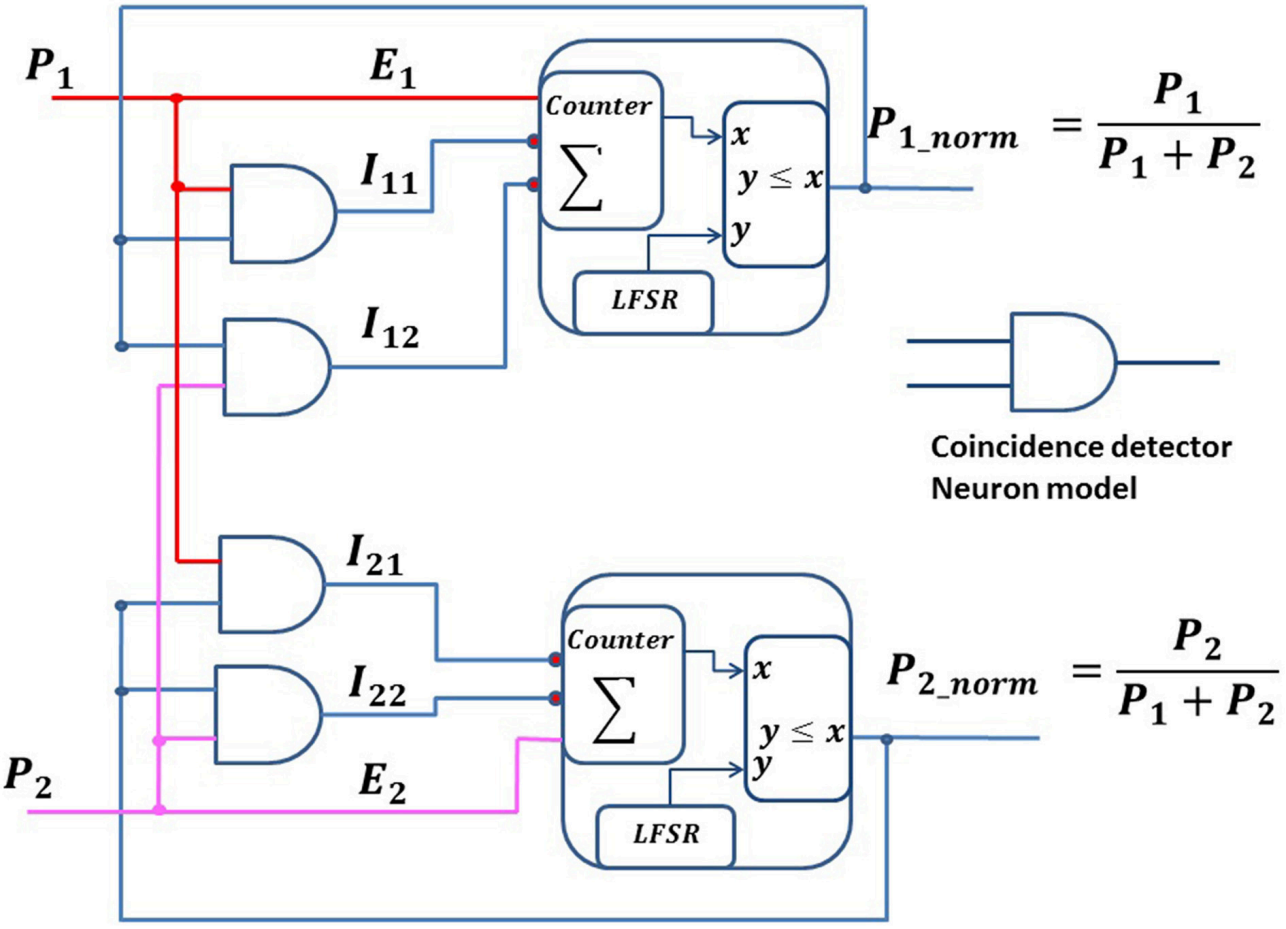
**Normalization neuronal circuits**. The schematic shows two normalization circuits that normalize the spike train inputs, *P*_1_ and *P*_2_.

The DNC can be generalized for multiple excitatory and inhibition connections. We have developed a normalization (*norm*) neural circuit by connecting an additional inhibition input to a DNC. The *norm* neural circuit computes a normalization of the firing rate of the input neurons in the spike domain. Figure 6 shows two *norm* neural circuits, which generate spike trains with a rate equal to their input firing rate divided by the sum of the input firing rates. Each *norm* neural circuit is a combination of an Inhomogeneous Poisson Neuron (IPN) and two CD neurons. Each IPN has one excitatory input, *E*_*i*_, and two inhibitory inputs, *I*_*i*1_ & *I*_*i*2_ (*here, i* ∈ 1, 2), coming from two CD neurons. These CD neurons receive spike inputs from the output of the IPN neurons (recurrent connections) and from the presynaptic neurons (forward connections). The membrane voltage (ρ) of an IPN is integrated using a saturating counter, which increases by one unit upon receiving an excitatory spike, decreases by one unit if either of the inhibitory inputs receives a spike, and decreases by two units if both the inhibitory inputs receive spikes. The IPN generates a spike based on its instantaneous membrane voltage (ρ) at each time step. Similar to DNC circuit, we can write the equations for the *norm* neural circuit as below:

$$\begin{array}{l} E_{1}=P_{1};E_{2}\;=\;P_{2} \end{array}$$

where, *E*_1_ and *E*_2_ represent excitatory synaptic inputs to the *norm* neural circuits, and *P*_1_ and *P*_2_ represent the firing rates of the presynaptic neurons. The output spike from the norm neural circuit and presynaptic spike are independent of each other, thus the average firing rate of the CD neuron would be the multiplication of the firing rate of its inputs.

$$\begin{array}{l} I_{11}=P_{1\text{\_}norm}\;*\;P_{1}\\ I_{12}=P_{1\text{\_}norm}\;*\;P_{2}\\ I_{21}=P_{2\text{\_}norm}\;*\;P_{1}\\ I_{22}=P_{2\text{\_}norm}\;*\;P_{2} \end{array}$$

where, *I*_11_, *I*_12_, *I*_21_, and *I*_22_ are outputs of the CD neurons and represent inhibitory connections to the *norm* neural circuits. *P*_1*_norm*_ and *P*_2*_norm*_ are the output firing rates of the *norm* neural circuits.

At equilibrium, the excitatory input and the inhibitory inputs from a neuron are equal. Thus,

(6) $$\begin{array}{l} E_{1}=I_{11}+I_{12}\\ P_{1}=P_{1\text{\_}norm}\;*\;P_{1}+\;P_{1\text{\_}norm}\;*\;P_{2}\\ P_{1\text{\_}norm}=\frac{P_{1}}{P_{1}+P_{2}}\; \end{array}$$

Similarly,

(7) $$\begin{array}{l} P_{2\text{\_}norm}=\frac{P_{2}}{P_{1}+P_{2}} \end{array}$$

The output of the *norm* neural circuits thus computes the normalized version of its input firing rate as demonstrated in Equations (6) and (7), as long as *P*_1_ and *P*_2_ vary more slowly than the time it takes for the *norm* neural circuits to reach their equilibrium state.

##### Stochastic exponential moving average (SEMA)

**Figure 7:**
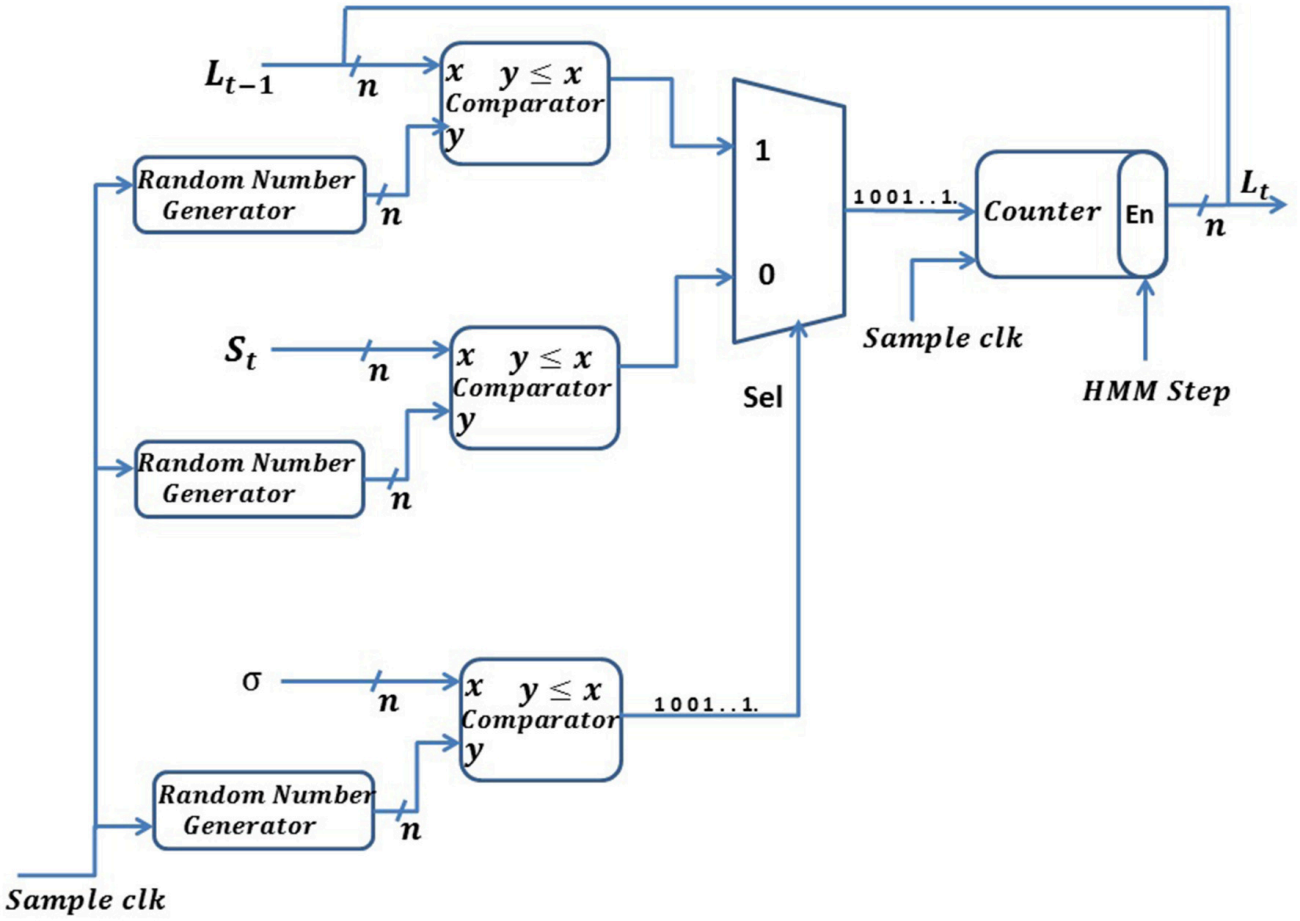
**Schematic of the stochastic exponential moving average (SEMA) filter**.

An exponential moving average filter is used for smoothing time series input data. It is a type of a recursive low pass filter, which can be described by Equation (8).

(8) $$\begin{array}{l} L_{t}=\;\sigma \;*\;L_{t-1}+\left(1-\sigma \right)\;*\;S_{t} \end{array}$$

where, σ is the smoothing parameter, and 0 < σ < 1, but σ is generally close to 1, which will make the output *L*_*t*_ respond more slowly to a change in the input samples, *S*_*t*_. We have used σ equal to 0.99 for all our simulations below. *L*_*t*_ represents the current average of the series as estimated from the data up to the present and *S*_*t*_ represents the current observation. The value of *L* at any given time is calculated from its previous value. We have implemented a stochastic version of the exponential moving average, which we refer to as the SEMA (stochastic exponential moving average). In the SEMA filter, all the variables of Equation (8) are represented by spike trains. Implementation of the SEMA filter is shown in Figure 7. We convert all variables, of size *n* bits, into spike trains using a random number generator circuit implemented using the commonly used *LFSR* (Linear Feedback Shift Register) circuit (Golomb et al., 1982). The smoothing constant, σ, is also represented with *n* bits precision and normalized between [0, 2^*n*^−1]. Thus, in Equation (8), (1−σ) is replaced by (2^*n*^−σ). In our implementation, we define two different time domains: HMM time steps and system clock time steps. One HMM time step comprises of 2^10^ system clock steps. We explain the different time domains in further detail in Section Hardware Implementation of the BEAST Framework.

##### Linear feedback shift register (LFSR)

An LFSR is a shift register whose input bit is a linear function of its previous state. Some of the outputs of the shift registers are combined using XOR gates to form the feedback mechanism. Here, we have used the maximum length sequence in an LFSR, which generates 2^*n*^−1 random numbers before it repeats itself, where *n* is the number of bits in the shift register. The feedback points in the shift register (called taps) are chosen based upon a characteristic polynomial for that number of taps to ensure a maximum length sequence. The list of taps for the maximum length LFSR have been published previously (Alfke, 1996). An LFSR is known to be a very simple, but not very good random number generator. The output sequence of an LFSR is not truly random and the output streams can be determined if the initial seed and the position of the XOR gates is known. Another concern is the periodicity of an LFSR, where the output sequence repeats itself after achieving the maximum length. Short repetition periods would affect the performance of the system because it would result in high correlation among the bit streams. This can be minimized by using a large *n* for the LFSR. In our frameworks the key concern is to ensure minimal correlation between the bit streams that are inputs to the stochastic elements such as the AND gate. As all our internal variables are 8 bits in size, a 10-bit LFSR of which we only select 8 bits for comparison, will generate random numbers with a large enough period to ensure there is no correlation among the bit streams of the variables.

#### Hardware implementation of the BEAST framework

We consider *M* sensory neurons and their corresponding *M* inference neuronal circuits, which include the likelihood, posterior and prior blocks in our BEAST framework as shown in Figure 2, and define an HMM time step corresponding to each step of the fruit fly. In one HMM step, sensory neurons will operate only once, while all the inference circuits will operate *N* times. An HMM time step of size Δ divided into *N* time bins defines the hardware system clock as Δ/*N*. In this version, we can estimate the posterior distribution from the spike counts. Such a neural implementation requires few neurons, but needs a long period of time (*N*) to compute the spike count. An alternative implementation is also possible, where there are *N* inference neuronal circuits corresponding to the receptive field of each sensory neuron, and the inference circuit operates in the same HMM time step. This alternate implementation would be more biologically plausible, because the neurons do not need to spike faster than the HMM time step. However, this would need M^*^N inference neuronal circuits, which would require a large area to implement in hardware. We define that the nervous system of our virtual dragonfly operates at the time scale of milliseconds, and we have used *N* equal to 1024 (2^∧^10). This will constrain our system clock to be no slower than ~1 MHz. The typical clock frequency of an FPGA (Field Programmable Gate Array, the hardware platform on which we implement our BEAST framework), is hundreds of MHz and thus we can easily meet the timing restrictions imposed by the dragonfly's nervous system. We have chosen the number of sensory neurons, *M*, as 17 for our implementation because we need to divide by (*M*–1) during the learning of the distractor probability (see Appendix 2 in Supplementary Material). Division by 16 can be easily implemented in hardware using a shift operation. All pathways are implemented in parallel on the FPGA hardware.

We will first show the hardware implementation of the model to perform the Bayesian inference of the hidden state, assuming all parameters are known. Next, we will show how we can estimate the various parameters of the model such as the transition probability, the observation probability and the distractor (background noise) probability, and their hardware implementation.

##### Inference

We present an algorithm similar to the SMC technique to compute the posterior probabilities of the state space to estimate the fruit fly position. SMC is a Monte Carlo technique useful for sequential Bayesian inference (Gordon, 1993). In the BEAST framework (Figure 2), spikes are used to represent a probability distribution over some set of states, such that the expected sum of the spikes in any state is proportional to the probability of that state. For example, if the posterior of a state is represented by a train of 256 spikes in one HMM time step (2^∧^10 clock cycles), then the posterior probability of that state is 0.25. As shown in Figure 2, the RS (resampling) neuron block has spatial connections which encode the transition model, P(*f*_*t*_ |*f*_*t*−1_), to estimate the fruit fly position. The three basic building blocks of the implementation are the *likelihood generator block*, the *posterior generator block*, and the *prior generator block*. These blocks are described in detail below. The pseudocode for the model's implementation is provided in Appendix 1 of Supplementary Material.

The *likelihood generator block* (green boxes, Figure 2) has a Poisson neuron (PN), which generates spike trains based on its intrinsic firing rate (α, or αβ as indicated in brackets in Figure 2). For each discrete fly location, *i*, there are two PNs for the two likelihoods, *L*_1*i*_ and *L*_0*i*_, because the dragon fly does not know if a sensory spike is due to a fly or due to background activity. For a sensory spike at location, *i*, PN(*L*_1*i*_) encodes for the potential presence of fly and it fires with probability α, independent of what is in its receptive field background, and spike trains are generated to encode probability α. At the same time, PN(*L*_0*i*_) encodes the potential presence of a distractor and if there is no fly in the afferent's receptive field, then there is a distractor in the receptive field with probability β, and spike trains are generated by PN(*L*_0*i*_) to encode the probability αβ. In the absence of a spike from the *ith* afferent neuron, a PN(*L*_1*i*_) generates spike trains to encode the probability P(*S*_*k*_ = 0|*f*_*k*_) = 1−α, and another PN(*L*_0*i*_) generates spike trains to encode the probability P(*S*_*k*_ = 0|*f*_*k*_) = 1−αβ. These can be interpreted as the spontaneous rates of the PNs, i.e., in absence of any stimulus.

The *posterior generator block*, consisting of the coincidence detector (CD) neurons and the normalization (*norm*) neural circuit, is shown in orange in Figure 2. According to Bayes rule, the posterior function is proportional to the product of the likelihood function and the prior function. As the likelihood spike train is independent of the prior spike train, the posterior can be implemented using a simple *AND* logic gate as an abstract model of the CD neuron. The output spike trains of the CD neurons are un-normalized posterior probabilities of not having a fly and having a fly in the receptive field, respectively, which are passed to the *norm* neural circuit to generate normalized spike trains.

The *prior generator block* is shown in magenta in Figure 2. Recurrent connections in the BEAST framework are from adjacent *norm* neural circuits and from the *norm* neural circuit of the same pathway. The recurrent connection weights are based on the transition probabilities. We have shown these connections in Figure 2 using red, orange and purple arrows. For the sake of simplicity in the BEAST framework, we have considered pathways *n* and 1 to be adjacent to each other, but in reality one could implement explicit border conditions for the array. As shown in Figure 2, the *RS* (resampling) block for the *i*th pathway samples spikes from the posterior distributions of adjacent *norm* neural circuits, *i*–1, *i* and *i*+1, according to their transition probabilities. In hardware implementation, the prior probability of each state is calculated by sampling from the adjacent pathway using an inverse transform sampling method in a discrete distribution. This method works as follows:

1. Generate a random number from a uniform distribution in the interval [0,1].
2. Check where this random number falls in a cumulative distribution. For example, if we have a transition probability of (0.2, 0.1, 0.7) for (*Left, Stay, Right*) in the BEAST framework, this gives a cumulative distribution of (0.2, 0.3, 1). We resample from adjacent distributions (*i*–1, *i* and *i*+1) based on which interval of the cumulative distribution function the random number falls in.
3. Repeat steps 1 and 2 for *N* times.

The *winner-take-all* (WTA) circuit in Figure 2 computes the maximum a posteriori (MAP) estimate of the probability distribution across states. The output of the WTA circuit is the most probable fruit fly position at time *t*.

##### Learning

In the previous section, we have assumed that the model parameters—transition probabilities, observation probabilities and distractor probabilities, are known. In this section, we describe how these parameters can be estimated from noisy observations and how these can be implemented using very simple digital circuits. In the BEAST framework, the dragonfly first learns the observation model parameters, α and αβ, under static conditions assuming the fruit fly is not moving. We do not calculate β as a separate parameter, because in our model we do not use β explicitly, but it is always used as the αβ term. Then, we relax the static constraints and the dragonfly learns the transition probability of the moving fruit fly in a dynamic environment.

The observation model parameters, α and αβ, are estimated by observing spikes of the sensory neurons and collecting statistics of the spikes over many HMM time steps. During learning of the observation model, we assume that there is only one fly and it is not moving. Thus, in each HMM time step, a sensory spike will be generated with probability α at the location corresponding to the fly position, and with probability αβ at all other locations. We initialize parameters α and αβ at the start of the learning process, and update these at each HMM time step based on a spike (or no spike) for each location using stochastic exponential moving average filters (Section Neuronal Building Blocks). At each HMM time step, the maximum value of all SEMA filters represents an estimated α and the remaining values are averaged to represent αβ. For the next HMM time step, these new values of the parameters are used. After collecting a sufficient number of spikes, the output of each SEMA filter converges toward the true value of the probability for each location. As the fly is not moving, the location corresponding to the fly position in the receptive field will converge to a probability α and the other locations will converge to probability αβ. The pseudocode for the estimation of this parameter is provided in Appendix 2 of Supplementary Material.

The transition probabilities are calculated based on the estimated location of the fly in the previous and the current HMM time steps. In our model, the fruit fly can move only one location in one HMM time step, which is effectively saying that the neural system estimates positions faster than the fruit fly can move. The transition probability (trans_prob vector in the pseudocode in Appendix 3 of Supplementary Material) of each direction is updated based on the direction the fruit fly moves in each HMM step, and smoothed using the SEMA filter described in Section Neuronal Building Blocks. At each HMM time step, we calculate the difference between the current estimated location and the previous estimated location (say, variable *delta*). The possible values of *delta* are (−1,0,1), which represent the movement in (Left, Stay, Right) directions, respectively. We initialize the probability of fly movement in each direction [L,S,R] to be equally likely, i.e., 1/3 in each direction, and these probabilities are updated at each HMM time step based on the value of *delta* using SEMA filters (3 filters, one for each direction). For instance, if *delta* is 1, at an HMM time step, then right probability [R] will be increased and other two [L,S] will be decayed from their previous value. For the next HMM time step, these new values of the probabilities are used. After collecting sufficient statistics of the fly movement, the output of each SEMA filter converges toward the true value of the transition probability in each direction. We do not update transition probability when the absolute value of *delta* is more than one, because it indicates that the estimated value of the fly location is not correct either in the current or the previous time step. The pseudocode for the estimation of this parameter is provided in Appendix 3 of Supplementary Material.

#### Hardware implementation of the BIND framework

The BIND framework and its possible neural implementation are described in Figure 3. Here, we show that a network of stochastic spiking neurons can perform the probabilistic inference using sampling. Each random variable (V, A, F, M, C) is implemented as a PN, and the connection weights are determined by their probability distribution, which are described in a tabular form in Figure 3A. The coincidence detection of spikes from the PNs can be modeled as CD neurons (König et al., 1996) or as active dendrites (Softky, 1994; London and Häusser, 2005). Here, we consider each random variable as binary, which can be generalized to many possible values. In Figure 3B, these various probabilities (shown in orange) can be treated as synaptic connections among the neurons, and each synaptic connection is associated with the emission probabilities of a spike, thus the non-spiking probability would be one minus the spiking probability.

In the neural circuit of Figure 3B, the probability of a variable can be inferred from the multivariate joint distribution by estimating the spike rate of the corresponding PN. The steps describing how the neural sampling works are detailed in Section Bayesian INference in DAG (BIND) Framework. This can be achieved by taking multiple samples and only considering those samples where a spike is generated in the PN of the corresponding variable. This calculation also requires the division operation (Section Bayesian INference in DAG (BIND) Framework), which can be implemented using the DNC (Figure 6). In Figure 3B, we have also shown a division neural circuit to infer the probability of variable A given F and V. Similarly, we can build all possible neural circuits to infer the probability of any variable dependent on other variables.

## Results

### BEAST framework

We have implemented the BEAST framework both in software (MATLAB), and in hardware on the FPGA. The hardware implementation is exactly similar to its software version, except for the random number generation logic. In hardware, we have used an LFSR circuit, but in software the built-in random number generation function is used to speed up simulation. This difference has no noticeable effect on system performance and all results presented here are thus from the software version, due to the ease of simulating the various parameter ranges and collecting the results. We have also implemented the Bayesian recursive equations using floating point representations and compared the tracking accuracy with that of our stochastic computation (SC) framework. We have simulated the system for 50 HMM time steps. We have created a *flymov* function, which emulates the fly movement based on its transition probability and generates the environmental noise with probability β. We initialize the fly position from a random position in the receptive field of the dragonfly. Based on the transition probability in each time step, the fly moves either to the left, to the right or stays at the same position compared to its previous position. A binary noise event is added at each position of the receptive field with probability β. The resulting vector is used as the input to the BEAST system. At each time step, the dragonfly estimates the true position of the fly based on the internal model of the fly, modeled as an HMM framework. All the states in the receptive field of the dragonfly are arranged in a parallel neural path. At each HMM time step, the dragonfly receives noisy observations about the fly for each path, which passes through the sensory neuron (Likelihood), CD neuron (Posterior), and the *norm* circuit followed by the RS circuit (Prior). At the end of each HMM step, the maximum charge stored in the *norm* circuit (value of the counter) denotes the MAP estimate of the fly position. The bit size of the counter, which is used inside the *norm* circuit, has a bit width size of 8. We have used 2^10^ clock cycles to simulate one HMM time step. The observation and transition probabilities used during the inference and the learning process are mentioned in the following sections.

#### Inference in the BEAST framework

**Figure 8:**
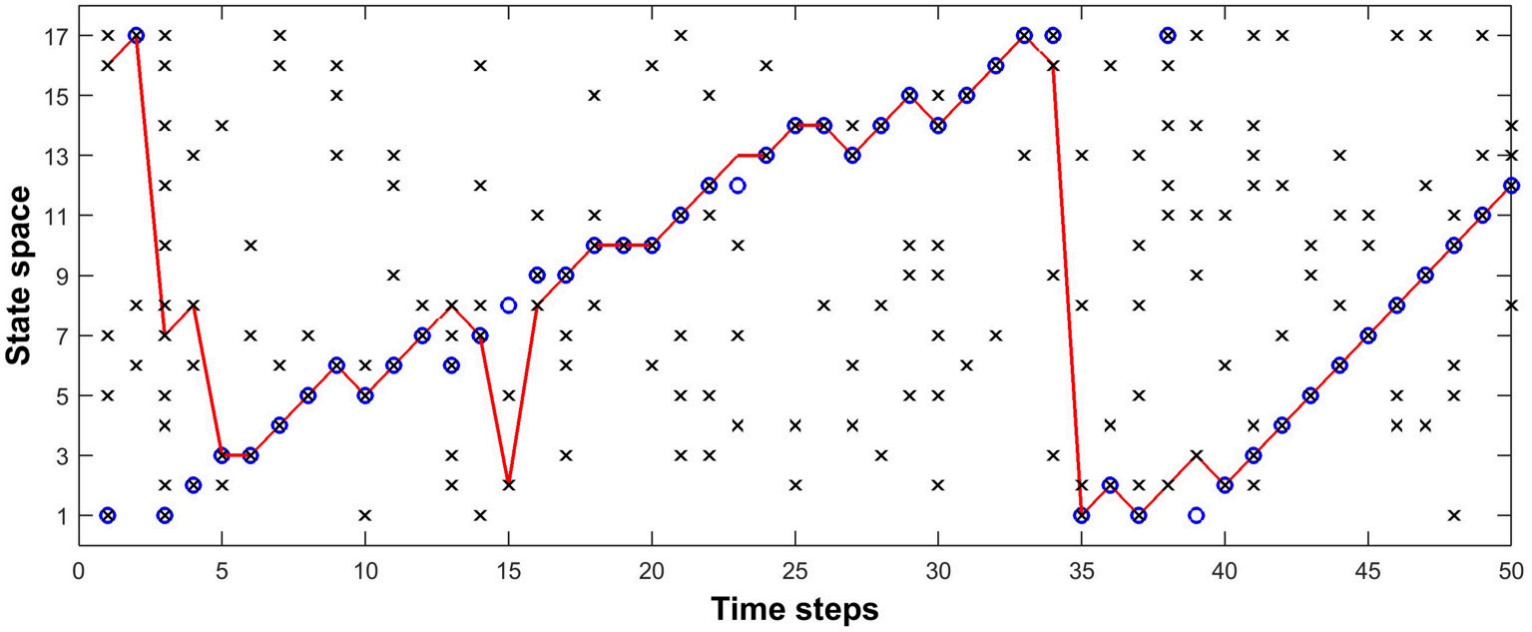
**Model tracking results**. A fruit fly moves against a randomly flickering background (β = 0.2). The sensory afferent neurons of the dragonfly fire probabilistically (α = 0.9) if there is a fruit-fly in the foreground or a false target in the background of their receptive field, and the presence of a spike at each sensory neuron in either case is represented by “x.” Inference neurons compute the posterior probability for each possible state using a given transition probability—in the example we use (0.2, 0.1, 0.7) for (*Left, Stay, Right*)—and a winner-take-all circuit determines the maximum *a posteriori* estimate of the fly's position at each time, shown by the red curve. “⊗” represents the fly position detected successfully by a sensory neuron, while “O” represents failure of the sensory neurons to detect the fly. This simulation shows that the dragonfly tracks the fruit fly even in the presence of distractors, with a prediction accuracy of 80% using the above-mentioned parameters.

Figure 8 shows the tracking results obtained using a fixed value of parameters. In Figure 8, blue circles represent the movement of the fruit fly, while the red curve represents the dragonfly's estimate of the fruit fly's position. In this simulation we have used 17 possible states in the receptive field of the dragonfly, and at each time step, the fruit fly is likely to be in one of those states. The probability of spikes when there is a fruit fly in a given state, α, was chosen to be 0.9 and the noise (distraction) probability, β, was chosen to be 0.2. The transition probabilities corresponding to [*Left, Stay, Right*] were *[0.2, 0.1, 0.7*]. The sensory afferent neurons of the dragonfly fired probabilistically (α = 0.9) if there was a fruit-fly in the foreground or a false target in the background of their receptive field, and the presence of a spike at each sensory neuron in either case is represented by black crosses “x.” In Figure 8, the simulation shows that the dragonfly tracks the fruit fly in the presence of multiple distractors, with a prediction accuracy of 80% (i.e., the dragonfly (red curve) tracks the fruit fly (blue circle) 40 times out of a total of 50 time steps) using the parameters α, β and the transition probabilities as 0.2, 0.9 and [*0.2, 0.1, 0.7*] respectively.

**Figure 9:**
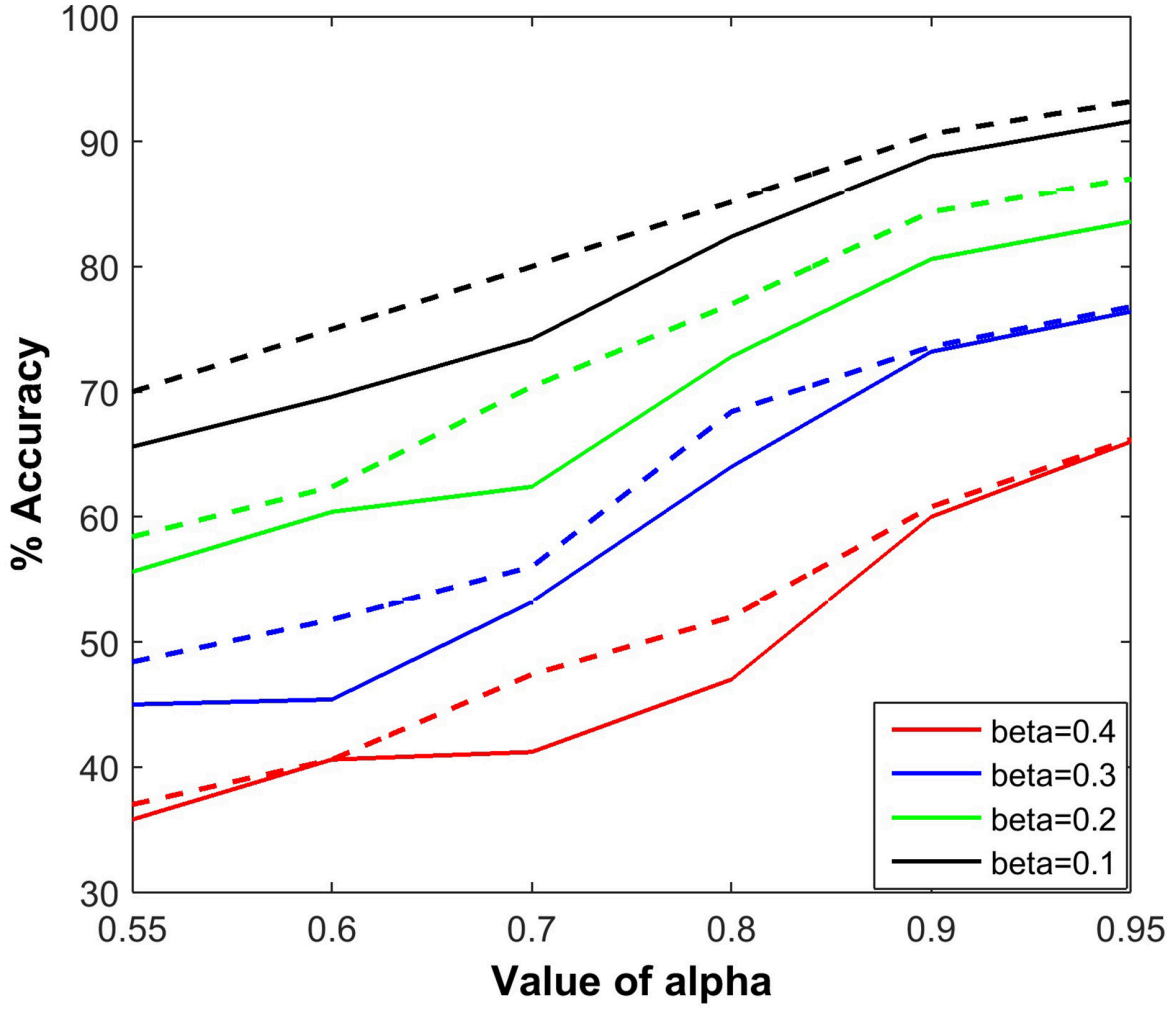
**Tracking accuracy of the BEAST framework as a function of α and β**. Each point is calculated by taking the mean of 10 different simulations. The probability of background activity, i.e., the presence of distractor is β and the probability of firing of the sensory neuron, either due to a fruit fly or a distractor is α. The performance results are compared for both types of implementations, using stochastic computation framework and using floating point implementation (dotted line).

We measured the effects of varying the parameters, α and β, on the accuracy of estimation of a fruit fly position using BEAST. Figure 9 shows the estimation accuracy for six different values of α and four different values of β, and compares the SC implementation with the floating point version (dotted line). For direct comparison, we have performed the simulations with the same random seed for both implementations. The tracking accuracy in the floating point implementation is slightly better than using SC, due to the variance in the stochastic representation. Each point in the figure is calculated by taking the mean of 10 different simulations. It is evident that increasing the noise (distraction) probability β negatively influences the tracking capability of the dragonfly by degrading the estimation accuracy. The estimation accuracy is also degraded as α decreases, indicating that imperfect propagation of information from the sensory neurons affects the tracking capability of the dragonfly.

#### Learning in the BEAST framework

**Figure 10:**
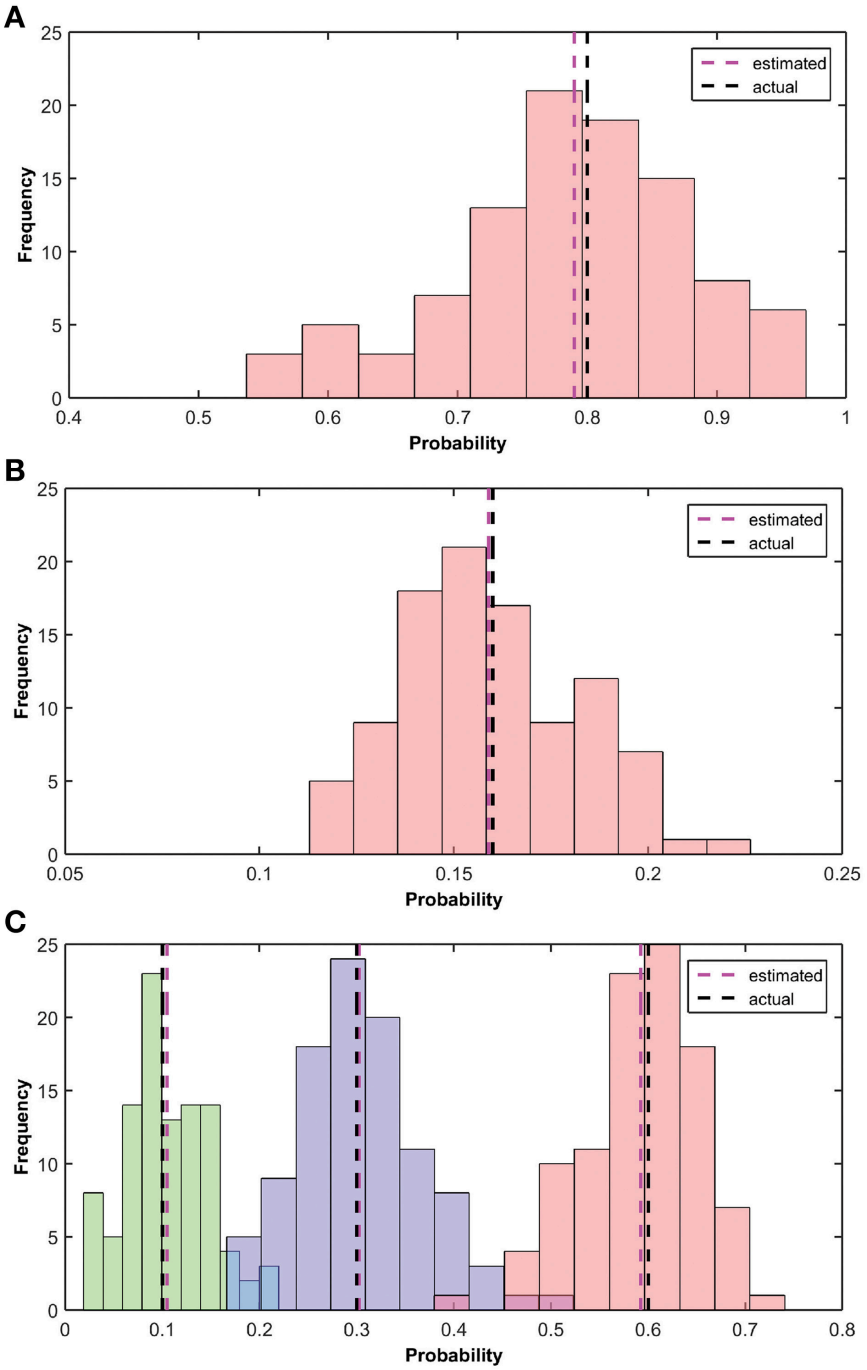
**Estimation of α, β and the transitional probability. (A)** Histogram of the estimated observation probability (α) is shown using pink bars. True (0.8) and average estimated (0.79) observation probabilities are shown using black and pink dotted lines, respectively. **(B)** Histogram of the estimated parameter αβ is shown using pink bars. True (0.16) and estimated (mean) parameter αβ (0.159) are shown using black and pink dotted lines, respectively. **(C)** Histogram of the estimated transition probabilities [Left, Stay, Right] of 100 different simulations are shown in [Green, Blue, Pink], respectively. True transition probability [0.1, 0.3, 0.6] and estimated (mean) transition probability [0.105, 0.302, 0.59] are shown using black and pink dotted lines, respectively.

In this section, we show the learning results of the various parameters of the BEAST framework. These parameters are learnt independently in the model. The implementation of learning is described in Section Hardware Implementation of the BEAST Framework. We have performed the simulation 100 times and calculated the mean of the results of all the simulations. Figure 10 shows the estimation of α, αβ and the transition probabilities. In our simulation, the fruit fly was moving [Left, Stay, Right] with actual transition probabilities of [0.1, 0.3, 0.6], β was 0.2, and α was 0.8, i.e., αβ was 0.16. We initialized the simulation with a transition probability estimate of [0.333, 0.333, 0.334], α of 0.6 and β of 0.1. The estimated values of the transition probability, α and αβ at the end of the simulation converged to [0.105, 0.302, 0.59], 0.79, 0.159.

### Inference results of the BIND framework

Here, we have compared inference of a few variables conditioned on other variables in the BIND framework, obtained through our SSM for different numbers of samples (mean and standard deviation of 30 results) to those calculated analytically (Table 1). As the number of samples increases, the variance in the result becomes smaller. We have represented random variables as n bits, which will determine the number of samples as 2^*n*^. It can be seen that the result using SSM is similar to the analytical solution, however, the analytical calculation of a probability distribution in a multi-dimensional space is computationally very expensive. The SSM requires only the very simple building blocks presented above to implement Bayesian networks on an IC, and is thus very efficient in terms of silicon area.

**Table 1:** **Comparison of the probabilities of the BIND framework (Figure 3), calculated using the SSM for various samples and the analytical methods**.

**Probability**	**Analytical**	**Stochastic sampling method (SSM)**		
		**2^∧^13 Samples (8192)**	**2^∧^12 Samples (4096)**	**2^∧^10 Samples (1024)**
P(C)	0.368	0.367 ± 0.005	0.368 ± 0.007	0.3701 ± 0.015
P(C|V)	0.555	0.554 ± 0.009	0.556 ± 0.013	0.564 ± 0.025
P(A|F)	0.437	0.438 ± 0.009	0.436 ± 0.014	0.431 ± 0.029
P(A|F,V)	0.391	0.391 ± 0.010	0.389 ± 0.014	0.386 ± 0.029
P(V|F,M)	0.988	0.987 ± 0.003	0.987 ± 0.04	0.987 ± 0.009

## Discussion

We have presented Stochastic Computation building blocks for Bayesian Inference which can be very simply implemented in hardware. With these building blocks, we have implemented a simplified fly tracking algorithm using the BEAST framework, which approximates Bayesian filtering using spikes as Monte Carlo samples of probability distributions. In our BEAST framework, first we have assumed that the transition probabilities and the observation models (α, β) are known and shown the tracking performance of the dragonfly corresponding to these parameters. We have also shown how the model parameters can be learnt and implemented using simple digital gates. Further, the posterior distribution of all the possible fly states is encoded as a sampled distribution represented by spikes across the inference neural population. The BEAST framework is based on an HMM, wherein sensory neurons of a dragonfly make noisy observations of external fruit fly positions at discrete time steps to predict the future location of a fruit fly, and update their belief each time a new observation is made. Here, we have presented neural circuits, and shown how these can be easily implemented using simple digital logic gates. We have implemented the BEAST framework on hardware and shown its capability to perform computations in real time.

We have also used the building blocks to implement inference in a probabilistic graph using SSM (the BIND framework). Implementation of this kind of sampling method on hardware is very area efficient and massively parallel systems can be built to run in real time. Our work demonstrates that complex probabilistic algorithms such as discrete HMM and DAG can be implemented in hardware, and can perform the computation in real time.

There is increasing evidence in neuroscience that estimation and inference in the brain is similar to Bayesian Inference. Thus, it would be helpful for neuroscience community if large biological plausible Bayesian models can be built on hardware to run in real time, which is difficult to simulate otherwise on a computer. Probabilistic graphical models provide a powerful framework to represent complex real world scenarios by combining probabilities (Larrañaga and Moral, 2011), which can be inferred approximately using SSM in real time. One of the potential applications of these systems is in speech recognition, where we need to build hierarchical HMM for syllables and words. Since this requires parallel implementation, our approach would be suitable to implement it in hardware to run in real time.

For the BIND framework, we are targeting to build a compiler, which can convert any DAG network into Verilog code that can be ported on an FPGA to infer random variables based on the observed variables using the sampling method. The implementation of these frameworks using simple logic gates may pave the way for a new kind of circuit paradigm called stochastic electronics (Hamilton et al., 2014), which will use randomness for its computation and will be optimal for probabilistic algorithms.

## Author contributions

All authors listed, have made substantial, direct and intellectual contribution to the work, and approved it for publication.

### Conflict of interest statement

The authors declare that the research was conducted in the absence of any commercial or financial relationships that could be construed as a potential conflict of interest.
