# Supplementary material for: Bayesian Estimation and Inference Using Stochastic Electronics
Source: Front Neurosci. 2016 Mar 18;10:104. doi: 10.3389/fnins.2016.00104 (PMC4796016; doi:10.3389/fnins.2016.00104)
Supplement: Supplementary file 1 [file DataSheet1.docx]

**Appendix 1. Pseudocode of inference in the BEAST framework**

**@HMM time step**

**@system clock (N times)**

**for each j ϵ fibres //parallel implementation on hardware**

1. **generate likelihood spike using *Likelihood Generator Block (*section 2.2.2)**
2. **generate prior spike using PN neuron (section 2.2.1) based on sum_prior probability calculated in step 7**
3. **generate posterior spike using CD neurons (section 2.2.1)**
4. **normalise posteriors using norm neural circuit (section 2.2.1)**
5. **integrate posterior spike as sum_post using counter**
6. **generate prior spike by resampling posteriors of neighbouring fibres**
7. **integrate prior spike as sum_prior using counter, for next HMM time step**

**end**

**end**

**mappos = max(sum_post)**

**end**

Since our implementation uses digital logic gates, we represent the probability with a precision of $n$bits, and *N* will be$2^{n}$. This will map the probability between$[0, 2^{n}-1]$.

**Appendix 2. Pseudocode of learning emission and distractor probabilities in the BEAST framework**

In this pseudocode, **ab_est_v** represents the vector containing the spikes observed by the sensory neurons (fibres). At the end of the simulation, **a_est** and **ab_est** represent estimated value of *α* and *αβ*.

**@HMM time step**

**@system clock (N times)**

**for each j ϵ fibres //parallel implementation on hardware**

1. **use a_est_v calculated in previous HMM step**
2. **use SEMA circuit for smoothing spike counts**
3. **sum output spike of SEMA circuit in variable ab_est_v using counter**

**end**

**end**

**a_est = max(ab_est_v);**

**ab_est = (sum(a_est_v)-a_est)/(fibres-1);**

**end**

**Appendix 3. Pseudocode of learning transition probabilities in the BEAST framework**

In this pseudocode, *mappos* and *mapos_old* are current and previous estimations of the hidden state at an HMM time step.

**@HMM time step**

**delta = (mappos - mappos_old)**

**if (abs(delta) < 2)**

**@system clock (N times)**

**for each j ϵ fibres //parallel implementation on hardware**

1. **update transition probabilities (trans_est) based on value of delta (-1,0,1 w.r.t left/stay/right) using SEMA circuit**
2. **integrate each time spike from the SEMA circuit in variable sum_trans_est using counter**

**end**

**end**

**trans_est = sum_trans_est**

**end**

The transition probabilities are only updated when the absolute value of the variable *delta* in the pseudocode is less than 2, because fly can move only one step. This will minimise the false updating of the transition probabilities.
